# HLA-A24 and survivin: possibilities in therapeutic vaccination against cancer

**DOI:** 10.1186/1479-5876-4-38

**Published:** 2006-09-04

**Authors:** Mads Hald Andersen, Rikke B Soerensen, Jürgen C Becker, Per thor Straten

**Affiliations:** 1 Center for Cancer Immunotherapy (CCIT), Department of Hematology, Herlev University Hospital, Dk-2730 Herlev, Denmark; 2 Department of Dermatology, University of Würzburg, D-97080 Würzburg, Germany

## Abstract

Recently, it was described that an HLA-A24 restricted peptide derived from the survivin splice variant survivin-2B can be recognized by CD8(+) cytotoxic T-cells. The identification of an HLA-A24 epitope is critical for survivin-based immunotherapy as HLA-24 is the most frequent HLA allele in Asia. Consequently, this survivin-2B epitope is already a target in a clinical study in patients with advanced or recurrent colorectal cancer expressing survivin. However, the splice variant survivin-2B has been described to be pro-apoptotic, and is only expressed at low levels in most malignant tissues. Furthermore, survivin-2B expression are significantly decreased in later tumor stages and inversely correlated with tumor differentiation and invasion. Consequently, survivin is a more general vaccination candidate than the splice variant survivin-2B. Here, we on the basis of spontaneous immune responses in HLA-A24+ cancer patients describes that a HLA-A24-restricted survivin epitopes does indeed exist. Consequently, this epitope is an attractive target for the ongoing survivin-based peptide immunotherapy against cancer.

## Background

The group of Sato recently attempted to identify a HLA-A24-restricted epitope derived from the universal tumor antigen survivin. Although unsuccessful, they reported that an HLA-A24 restricted peptide (AYACNTSTL) derived from the survivin splice variant survivin-2B can be recognized by CD8(+) cytotoxic T-cells [1,2]. Subsequently, as described in this journal this survivin-2B epitope is the target in a phase I clinical study assessing the efficacy of survivin-2B peptide vaccination in patients with advanced or recurrent colorectal cancer expressing survivin [3].

As HLA-A24 is a very frequent allele expressed especially in the Asian population a survivin-derived HLA-A24 restricted epitope would be highly interesting in peptide based immunotherapy against cancer. Survivin is an apoptotic inhibitor that is expressed at high levels in a variety of malignancies. At present, four splicing variants are known for survivin (Survivin, Survivin-2B, Survivin-deltaEx3, Survivin-3B, and survivin-2α) [4-6]. Survivin is over-expressed in almost all cancers including lung, colon, breast, pancreas, stomach, liver, ovaries, and prostate cancer, as well as melanoma, and hematopoetic malignancies [7-9]. Data from a large analysis of human transcripts revealed survivin as the fourth most highly expressed protein in human cancer tissue compared to normal tissue [10]. In contrast, the expression and function in human cancer of the splice variant the survivin-2B is more indistinct. Thus, most studies describe survivin 2-B as to be pro-apoptotic, and only to be expressed at low levels in most malignant tissues [11]. Furthermore, survivin-2B expression are significantly decreased in later tumor stages and inversely correlated with tumor differentiation and invasion [4,11]. Likewise, Islam *et al *(2000) reported that the expression of survivin-2B is predominant in some neuroblastoma with a good prognosis [12], indicating a possible unfavourable role of survivin-2B in cancer development [13]. In contrast, two recent papers show that high expression of survivin-2B variant is correlated with poor prognosis in cancer patients [14,15]. However, as survivin-2B is not expressed in many malignancies, survivin is a more attractive candidate as a general vaccination candidate than the splice variant survivin-2B. In the present manuscript, we identified an HLA-A24 restricted epitope derived from the universal tumor antigen survivin.

## Materials and methods

### Patients

Peripheral blood lymphocytes (PBL) from HLA-A24 positive cancer patients were obtained from the University Hospital in Herlev, Denmark. The PBL were isolated using Lymphoprep separation and cryopreserved in FCS with 10% DMSO. Tissue typing was conducted at the Department of Clinical Immunology, The State Hospital, Copenhagen, Denmark. Informed consent was obtained from the patients before any of theses measures.

### Interferon-γ and perforin ELISPOT

The interferon-γ and Perforin ELISPOT assays was used to quantify peptide epitope-specific interferon-γ and perforin releasing effector cells, respectively as described previously [16,17]. Briefly, nitrocellulosebottomed 96-well plates (MultiScreen MAIP N45; Millipore) were coated with 7.5 μg/ml capture anti-human interferon-γ (Pf-80/164, Mabtech, Sweden) or 30 μg/ml coating anti-human perforin (Pf-80/164, Mabtech) in 100 μl sterile PBS per well. The wells were washed and T2-A24 cells (a kind from Yasuto Akiyama, Tokyo) and effector cells were added with or without 10 μM peptide. In some assays isolated, CD8 positive T-cells were used as effector cells using CD8+ MACS microbeads (Miltenyi Biotec GmbH, Gladbach, Germany). After incubation (37°C/5%CO_2_) medium was discarded and the wells were washed prior to addition of the secondary detection Ab, anti-human biotinylated interferon-γ (Mabtech) at 2 μg/ml in 75 μl PSB/BSA per well or anti-human biotinylated perforin (Pf-344-biotin, Mabtech) at 1 μg/ml in 100 μl PSB, respectively. The plates were incubated at RT for 2 h, washed, and 100 μl of streptavidin-ALP (Mabtech) diluted 1:1000 in PBS, was added to each well and the plates were incubated for one hour at RT and the enzyme substrate NBT/BCIP (Invitrogen Life Technologies) was added to each well. Upon appearance of dark spots, the reaction was terminated by washing with tap water. The spots were counted using the ImmunoSpot Series 2.0 Analyzer (CTL Analyzers).

## Results and discussion

**Figure 1:**
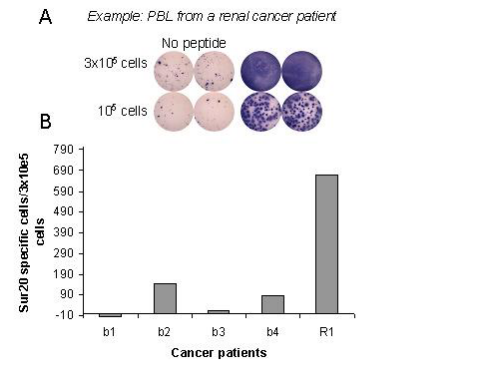
**HLA-A24 restricted T-cell responses against Sur20–28 in cancer patients**. A: Example of the ELISPOT plate performed with the strong responding renal cancer patient R1. B: Spontaneous T cell responses against Sur20–28 as measured by the ELISPOT assay. The average number of peptide specific IFNγ spots formed in response to Sur20–28 among 3 × 10^5 ^in vitro stimulated PBL from four breast cancer patients (b1, b2, b3, b4), and 1 renal cancer patient (R1). Non-specific IFNγ spots are subtracted.

**Figure 2:**
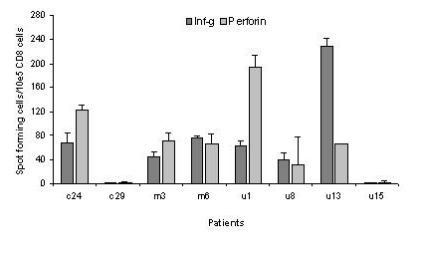
**Detection of HLA-A24 restricted, sur20–28 specific, CD8 postitive cells by interferon-γ and perforin**. ELISPOT. CD8+ cells were isolated from PBL from two HLA-A24+ breast cancer patients (c24, c29), two HLA-A24+ melanoma patients (m3, m6), and four HLA-A24+ renal cancer patients (u1, u8, u13, u15). Spontaneous T-cell responses against Sur20–28 was measured by both interferon-γ and perforin ELISPOT for all patients. The average number of peptide specific IFNγ or perforin spots formed in response to Sur20–28 among 10^5 ^in vitro stimulated CD8+ cells. Non-specific spots are subtracted.

Recently, a library of overlapping nonamers spanning the length of the survivin protein was used to screen for peptides capable of binding to different HLA alleles, including HLA-A*2402 revealing that the peptides Sur20–28 (STFKNWPFL), Sur96–104 (LTLGEFLKL), Sur133–141 (RAIEQLAAM), Sur126–135 (ETAKKVRRAI) bind to HLA-A24 [18]. These peptides were not included in the study from Sato and colleagues. Subsequently, we scrutinized PBL from HLA-A24+ cancer patients of different origin by means of ELISPOT against these peptides as described [19]. Indeed, strong and frequent CTL responses were detected against Sur20–28 (STFKNWPFL) in cancer patients of different origin as exemplified in figure 1a; an HLA-A24+ renal cancer patient and an HLA-A24+ breast cancer patient hosting strong spontaneous responses against Sur20–28. We were able to detect a response against Sur20–28 in three out of five examined cancer patients (figure 1). The IFN-γ ELISPOT assay is one of the most useful techniques for immunological monitoring of CTL responses and has gained increased application as a measure of specific T cell activation. Previously, we and others have identified CTL epitopes from e.g. survivin, Bcl-2, tyrosinase, CEA, EpCam and Mage on the basis of spontaneous T-cell responses in PBL from cancer patients as presented here [19-25]. However, the IFN-γ ELISPOT does not assess cell-mediated cytotoxicity directly as IFN-γ secretion is not limited to only cytolytic CD8 T-cells cells. Thus, although it has been shown that IFN-γ ELISPOT reactivity in most cases correlates with the capacity to exhibit cytotoxic function, the formal prove for this notion can only be obtained directly. Perforin is a key mediator of target cell death and the perforin ELISPOT assay was recently demonstrated to provide an estimation of cytotoxic effector cell frequency [17]. To insure that the ELISPOT positive cells were indeed CD8+ T-cells, we first isolated CD8+ cells from PBL from eight cancer patients. Subsequently, we analyzed these CD8+ cells both by IFN-γ and perforin ELISPOT (figure 2). In six out of eight patients we were able to detect not only an INF-γ response but in addition a perforin response. Furthermore, the CD8 isolated cells from the eight patients were tested against the irrelevant, HLA-A24-restricted epitope from HIV-1 gag gp41_67–75 _(RYLKDQQLL) [26]. Data from these experiments showed highly comparable results when testing unpulsed T2-A24 cells vs. T2-A24 cells pulsed with HIV-1 gag gp41_67–75_, even after a 10 day stimulation of the CD8+ cells with the HIV-1 gag gp41_67–75 _peptide (data not shown).

Thus, the Sur20–28 reacting cells in the patients PBL are indeed cytotoxic, CD8+ effector cells. Consequently, Sur20–28 is a very attractive target to be included in the ongoing survivin-based peptide immunotherapy against cancer.
